# Genetic regulation of leaf morphology in own-rooted and grafted vines of an F1 rootstock population

**DOI:** 10.3389/fpls.2025.1625453

**Published:** 2025-10-23

**Authors:** Prakriti Sharma, Dilmini Alahakoon, Jason P. Londo, Anne Fennell

**Affiliations:** ^1^ Agronomy, Horticulture and Plant Science, South Dakota State University, Brookings, SD, United States; ^2^ School of Plant Science, Horticulture Section, Cornell University, Geneva, NY, United States

**Keywords:** grapevine rootstocks, leaf area, Marquette, QTL, mapping, genetic regulation, *V.rupestris*, *V. riparia*

## Abstract

Understanding the genetic basis of leaf size and shape is essential for evaluating and selecting for plant adaptability and performance in variable and shifting climatic conditions. This study maps the leaf size and shape phenotypic variation as influenced by the genetic architecture of a rootstock population and its conferred influence on these traits in a common scion. The influence of the root system genotype was studied using two different presentations of an F1 rootstock population (F1_Vruprip; *V. rupestris* Scheele ‘B38’ (USDA PI#588160) X *V. riparia* Michx. ‘HP1’ (USDA PI#588271)); 1) the F1_Vruprip grapevine progeny on their own roots and 2) a F1_Vruprip cohort that was grafted with the common scion scion 'Marquette'. Three leaf positions (apical, middle, and basal) were sampled in both presentations at two timepoints in two consecutive growing seasons. A twenty-one-point leaf morphological landmark coordinate analysis was conducted, and ten leaf size and six derived shape phenotypes were used for QTL mapping. Genetic analysis identified five distinct hotspots associated with size-related leaf area attributes in own-rooted and grafted vines. The identification of multiple leaf-growth-associated pathways in these hotspot regions strengthened the correlation between genetics and phenotypic traits. Shape related QTL accounted for 12-48% of the shape phenotypic variation but did not cluster as QTL hotspots. Three QTL hotspots captured the genetic influence of the rootstock conferred onto the scion leaf area traits. The results showed that the leaf position and the rootstock population’s genetic composition significantly impacted leaf morphological attributes and that there was a measurable rootstock genotype influence conferred on the grafted scion leaves. This reveals the genetic loci and gene pathways underlying leaf morphological phenotypes in own-rooted progeny and also verifies the potential of rootstock genetics to confer modulation of scion canopy features, providing greater potential to select for climate-resilient grapevines.

## 1 Introduction

The morphology of the leaf blade and petiole and stomatal density, exhibit significant variability among plants (Bar and Ori, 2014; Chitwood and Sinha, 2016; Tsukaya, 2018). In addition to variability between individual cultivars, populations, and species, these parameters can also differ within the same genotype. The ultimate geometry of leaf structures is the result of striking a balance between the competing goals of maximizing energy intake and reducing the damage caused by environmental pressures respectively (Fritz et al., 2018). Environment, developmental stages, and genotype all exert influence on the morphology and functional attributes of leaves. Different leaf sizes and shapes have a substantial impact on how carbon, water, and energy transfer between plants and the environment. This, in turn, influences the rate at which photosynthesis occurs (Brito-Rocha et al., 2016; Malhado et al., 2009; Fonseca et al., 2000). Therefore, the study of leaf shape and size is critical for understanding plant adaptation, photosynthetic efficiency, and evolutionary biology, as well as for estimating and optimizing plant resilience and productivity in dynamically changing environments.

For *Vitis* species, a distinct field known as “ampelography” is dedicated to studying the morphological characteristics of the leaves (Rendu, 1854). In the field of ampelography, the term is derived from Greek for “vine” and “the process of measuring,” the homologous morphology that exists between grapevine leaves has long been documented and implemented for the purposes of classification and identification. Methodology has progressed from manual measurements (Galet, 1979) to a digital approach that employs scanned leaf images, landmarking specific traits, and meticulous statistical analysis of the traits (Chitwood et al., 2014b). In grapevines, multiple studies have utilized digital imaging and landmarking techniques to quantify the leaf morphometric traits (Chitwood, 2021; Chitwood et al., 2021; Demmings et al., 2019; Klein et al., 2017; Chitwood et al., 2016a; Chitwood et al., 2014b; Chitwood et al., 2014b). In contrast to the many publications on phenotypic plasticity of leaf morphological trait variation between grapevine species and cultivars, very few studies have addressed the underlying genetic mechanisms in grapevines (Demmings et al., 2019; Welter et al., 2007).

In viticulture, genetic studies on leaf shape and size are crucial as they lay the groundwork for future breeding programs, particularly for marker-assisted selection, aimed at manipulating leaf canopy traits in grapevines. Welter et al. (2007), identified 27 significant QTLs affecting leaf morphology using a population of *Vitis hybrid* 'Regent' x *V. vinifera* L. 'Lemberger' simple sequence repeat (SSR) genetic map. This study indicates specific sites across the genome as key determinants of leaf teeth shape and orientation of leaf veins. Demmings et al. (2019) studied *Vitis* populations, using finite traits collected as ampelographic measurements as well as principal components derived from general procrustes analysis to understand genetic mechanisms underlying for leaf shape variation contributed by combinations of *Vitis* sp*ecies*. Leaf morphology was mapped using five different own-rooted mapping families, 1) *V. vinifera* 'Chardonnay' × *V. cinerea* B9; 2) “Horizon” × *V. cinerea helleri* 'B9'; 3) 'Horizon' × Illinois 547-1; 4) ‘*V. rupestris* Scheele ‘B38’ × 'Horizon'; 5) *V. aestivalis* Michx. ‘Norton’ × *V. vinifera* L. ‘Cabernet Sauvignon’ using genotyping-by-sequencing single nucleotide polymorphism (SNP) marker based genetic maps. In this study, co-located QTL (hotspots) identified were frequently associated with chromosome 1, 8, and 18 and attributed to shape features like lobing. The multi-population study identified potential candidate genes within the QTL loci, such as the JAGGED gene, DELLA family genes, Wuschel-related homeobox 1,13 gene – WOX1, WOX13, cup-shaped cotyledon3 - CUC3, and BLADE-ON-PETIOLE2 (BOP2) genes, all of which are known to have a role in leaf morphogenesis and pattern formation. While these studies have documented genetic regulation of leaf shape lobed characteristics in own-rooted genotypes, much remains unknown about the genetic regulation of size and shape by position and seasonal stages, as well as whether genetic regulation is conferred to the grafted scion.

The objective of this study is to evaluate variations in leaf shape and size in two presentations of an interspecific F1 population cohort developed from a cross between *V. rupestris* Scheele ‘B38’ *X V. riparia* Michx. ‘HP1’ and to identify genetic regions associated with leaf morphological traits. Variation in leaf shape in own-rooted populations are noted; however, grapevine cultivars are commonly grafted onto rootstocks, and it is also important to determine the conferred influence of the rootstock genetics on the scion. Specifically this study 1) determined the shifts in size and shape dynamics attributed to decreasing daylength hours; 2) investigated the primary factors that contribute to variations in leaf size and shape within a rootstock population as influenced by developmental position and daylength 3) ascertain whether the F1_Vruprip rootstock population confers an effect on the common scion for leaf shape or size; and 4) identify particular quantitative trait loci hotspots and candidate pathways that are linked to leaf morphological QTLs.

## 2 Materials and methods

### 2.1 Rootstock population

Two presentations of an F1 rootstock population (*V. rupestris* Scheele ‘B38’ (USDA PI#588160) X *V. riparia* Michx. ‘HP1’ (USDA PI#588271)) were utilized for the objectives of this research (Bhattarai et al., 2021) 1) the F1 grapevine progeny on their own roots and 2) replicate F1 progeny grafted with the common scion 'Marquette'. The grapevines were grown in greenhouse under natural light and 25-30/20-25°C day/night temperature at South Dakota State University Brookings, SD (44.31°C N, 96.80°W) in 2021 and 2022 (vines were 3 and 4 years old, respectively). Ecodormant spur-pruned vines were removed from the cooler the first week of June, root pruned, and repotted in a growing medium consisting of soil, perlite, and peat (1:2:2 by volume). Vines were watered daily and fertilized bimonthly using a custom trickle irrigation system with one dripper in each pot. The vines were maintained with 5 spurs, and a single shoot was trained vertically from each spur. The study used 135 own-rooted genotypes for both years (2021 and 2022) and a total of 139 (2021) and 71 grafted vines (2022, note several grafted vines were moved to a field planting and not available for this study). Leaves were collected from each vine during the growing season at natural seasonal daylengths of 14 h and 13 h (mid-August and first week of September, **Supplementary Table 1**). All vines were actively growing and with no shoot tip senescence or periderm development present during this period. At each timepoint an apical, middle, and basal leaf was collected from the same shoot. A total of 2,880 leaves were collected and landmarked in the two years. To capture the full range of morphological and developmental variation along the grapevine shoot, we sampled three leaves per vine representing young, middle, and old positions. Grapevine leaves vary in shape systematically during development, for example young expanding leaves at the shoot apex have prominent veins and reduced blade area, while mature leaves at the base of the shoot exhibit distinct heteroblastic shape compared to mid-shoot leaves (Chitwood et al., 2016b). Additionally, grapevine leaves have been shown to follow conserved allometric scaling, with vein-to-blade ratios decreasing as leaves expand, such that young, middle, and old leaves capture the physiological extremes and intermediate states of this shape change (Chitwood et al., 2021). The leaf from the apical region was identified as the first unfolded expanding leaf where the leaf blade oriented perpendicular to the direction of shoot growth. The middle leaves were fully expanded and midway between tip and base of the shoot. The basal leaves originated from the first node closest to the point where the shoot emerges from spur on the vine. The collected leaves were placed in Ziplock plastic bags and stored at 4°C until scanned (24 h). A genotype label, calibration card and leaf set comprising apical, middle, and basal leaves from each vine, were scanned using a Plustek OpticPro A320E (Lakewood NJ, US) Flatbed Scanner.

### 2.2 Leaf landmarking

**Figure 1:**
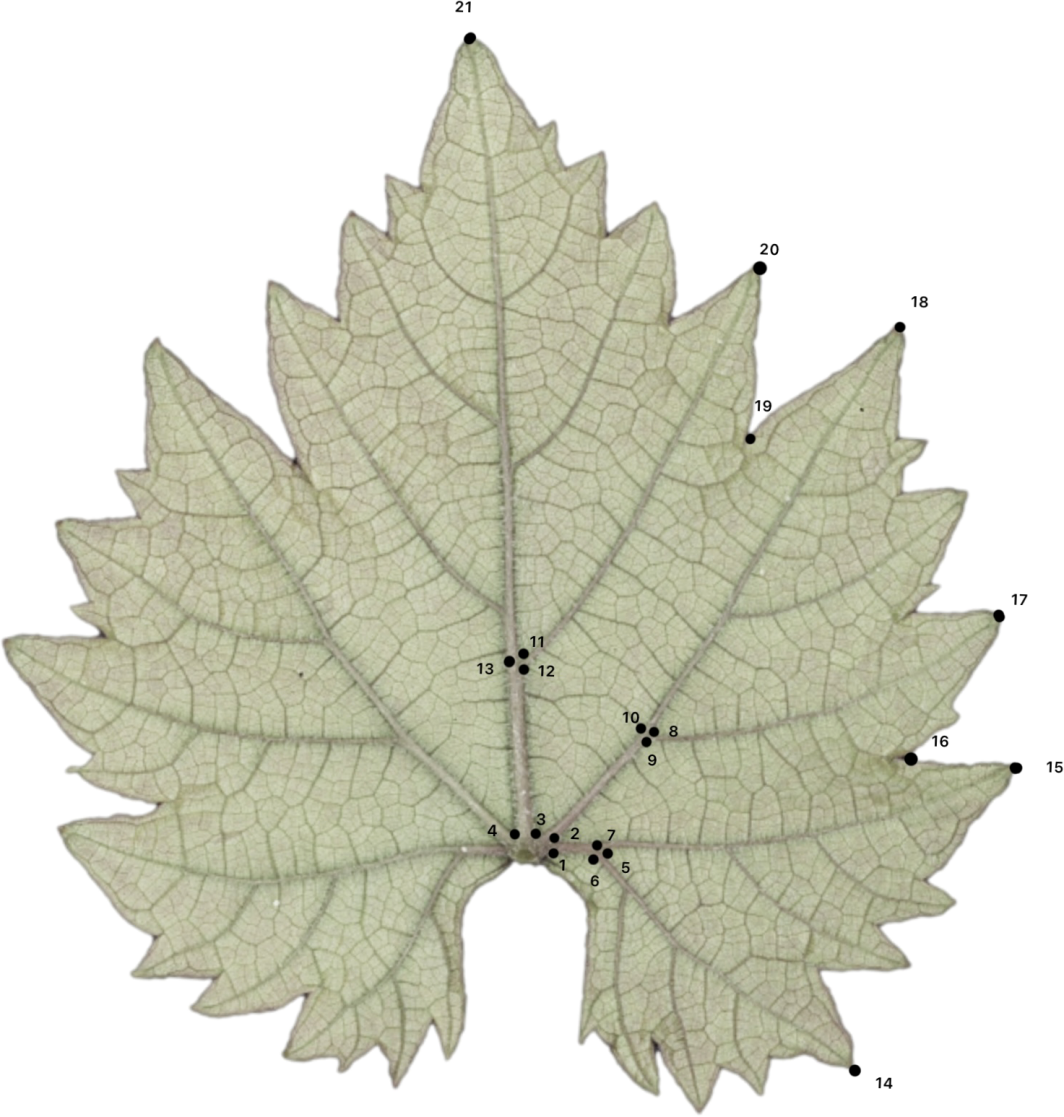
Illustration of 21 landmarks on the vine leaf. L1, L2, L3 and L4 are the measures of vein length in this study which refer to mid vein, distal vein, xvii vein and proximal vein, respectively. L5 is the measure of width of the petiolar sinus.

A total of 21 landmarks were manually labeled on the abaxial side of the images using Image J software (Abràmoff et al., 2004). As shown in **Figure 1**, these 21 landmarks consisted of: (1) left side of the proximal vein base, (2) right side of the proximal vein base/left side of the distal vein base, (3) right side of the distal vein base/left side of the midvein base, (4) right side of the midvein base, (5) distal base of petiolar vein, (6) proximal base of petiolar vein, (7) width of proximal vein at petiolar vein branch point, (8) distal base of distal vein branch, (9) proximal base of distal vein branch, (10) width of distal vein at branch point, (11) distal base of midvein branch, (12) proximal base of midvein branch, (13) width of midvein at branch point, (14) tip of petiolar vein, (15) tip of proximal lobe, (16) proximal sinus, (17) tip of distal vein branch, (18) tip of distal lobe, (19) distal sinus, (20) tip of midvein branch, and (21) tip of the leaf (Migicovsky et al., 2022; Chitwood et al., 2021; Bryson et al., 2020). To evaluate for position errors, ggplot in R (Wickham and Sievert, 2009) was used to replot the landmark coordinates into images, and images with errors were re-landmarked.

### 2.3 Morphometric analyses, statistics, and visualization statistics, and visualization

The ampelographic finite traits were derived using the 21 landmark coordinates. A total of 16 different phenotypes were obtained for each leaf sample, as outlined in **Table 1**. Distances between landmarks were also derived using the Pythagorean theorem (Maor, 2019). Ten phenotypes related to leaf size were computed, using the overall area of the leaf and the vein and blade area, utilizing the shoelace algorithm (Pure and Durrani, 2015). In addition, six different ratios were derived using area and length metrics as indirect measures describing proportion or pattern related to leaf shape.

**Table 1:** Size and shape metrics derived from 21 landmark coordinates and used in QTL analysis.

Characteristics	Traits	Description	Abbreviation	Ampelographic OIV designation
Leaf Size	Middle vein length	Length (L1) from midpoint of P3 and P4 to P21	mid_vein_length	OIV 601
	Proximal vein length	Length (L4) from midpoint of P1 and P2 to P15	prox_vein_length	OIV 603
	Distal vein length	Length (L2) from midpoint of P2 and P3 to P18	dist_vein_length	OIV 602
	XVII vein length	Length (L3) from P2 to P17	xvii_vein_length	
	Total area of leaf	polygon area defined by landmarks outlining leaf boundary	total_area	
	Blade area	difference between total leaf area and vein area	blade_area	OIV 065
	Vein area	sum of middle, proximal, and distal vein area	vein_area	
	Middle vein area	polygon area defined by middle vein landmarks	mid_vein_area	
	Proximal vein area	polygon area defined by proximal vein landmarks	prox_vein_area	
	Distal vein area	polygon area defined by distal vein landmarks	dist_vein_area	
Leaf Shape	Vein to Blade area ratio	Log of ratio (vein area/blade area)	veins_to_blade	
	Proximal to distal vein length ratio	ratio (L4/L2)	prox_to_dist	OIV 603/OIV 602
	Proximal to mid vein length ratio	ratio (L4/L1)	prox_to_mid	OIV 603/OIV 601
	Distal to mid vein length ratio	ratio (L2/L1)	dist_to_mid	OIV 602/OIV 601
	XVII to mid vein length ratio	ratio (L3/L1)	xvii_to_mid	
	Petiolar sinus length to leaf area ratio	ratio (petiolar sinus length (L5)/total area of leaf)	petiolar_sinus_to_area	

L1–5 are derived from coordinates in **Figure 1**. L1 (length from midpoint of 3 and 4 to 21), L2 (length midpoint 3 and 2 to 18), L3 (length from 2 to 17), L4 (length from midpoint 1 and 2 to 15), and L5 (cross length of the petiolar sinus). Formula’s for deriving the leaf size length and area values are found in **Supplementary Table 1**.

### 2.4 Descriptive statistics for morphometric traits

The Kruskal-Wallis test (McKight and Najab, 2010), a non-parametric approach was used to determine whether there were significant differences between treatment groups while comparing trait measures across time points or presentation types. The exploratory data analysis and visualization was performed in R utilizing ggplot and ggpubr (Kassambara, 2018). Pearson correlation analysis of traits was conducted using Pearson method and visualized using ‘ggcorrplot’ package in R. PCA was implemented using ‘prcomp’ builtin function in R to reduce dimension separating leaf morphological traits into size and shape categories. Relationship between distinct factors and phenotypic traits were explored generalized additive model (GAM) using ‘mgcv’ package in R. For descriptive analysis, the data were z-transformed to account for the scale differences associated with leaf positions and allow for standardized comparison in a unified figure. However, the original data were retained for QTL analysis preserving the accuracy of effect estimation.

### 2.5 Genetic linkage map and QTL analysis

#### 2.5.1 Genetic linkage map

Genotyping of 714 F1 offspring and their parents was conducted using 2000 rhAmpSeq core genome markers (Zou et al., 2020; Alahakoon et al., 2022). The linkage map was constructed using LEPMAP as previously described (Zou et al., 2020; Alahakoon et al., 2022). Of the 2000 markers used for genotyping, 1996 provided data. This yielded 133 singleton markers and identified homozygosity as an issue with 901 monomorphic markers. Allele-calling errors were checked prior to map construction and suspect loci were manually corrected. A logarithm of odds (LOD) of five was used to establish linkage groups and Kosambi map function were used for map distance (in centimorgans, cM) calculations. Linkage group orientation was corrected using the invert function if any inversions were found. Finally, the F1_Vruprip rhAmpSeq linkage map was formatted into R/qtl ABH format in MS Excel, where ‘A’ and ‘B’ allele, respectively, represent major and minor homozygous alleles and ‘H’ is the heterozygous allele. To evaluate the F1_Vruprip rhAmpSeq map, collinearity between the linkage map and the *V. vinifera* PN40024 12X V2 genome was measured by Spearman correlation coefficient (cor.test function in R) and visualized using correlation plot (ggplot function in R, **Supplementary Figure 1**). A pair-wise recombination fraction heat map was generated using plotRF function (qtl package in R) to evaluate marker order correctness (**Supplementary Figure 2**). The F1_Vruprip rhAmpSeq linkage map imaging was performed using MapChart (2.32 version) [67]. The QTL for flower type was used to validate the map (**Supplementary Figure 3**).

#### 2.5.2 QTL analysis

QTL mapping, a statistical method for identifying genomic regions associated with quantitative trait variation was performed using MetaPipe. MetaPipe (Villegas-Diaz et al., 2021) is a high-performance parallel processing pipeline that utilizes RQTL for large quantitative trait data set mapping. A total of 192 attributes, derived from the combination of 16 phenotypes, 3 leaf positions, 2 daylength, and a period of two years were mapped using F1_Vruprip rhAmpSeq. The total data matrix contained 25,920 and 20,160 genotype trait observations across two years for the own-rooted and grafted genotypes, respectively. MetaPipe was used to initially assess normality of each trait using the Shapiro-Wilk test and subsequently transform any non-normally distributed features to conform to normality assumptions. QTL mapping was conducted on normal traits using parametric methods and on traits that did not present as normal (ie skewed) using non-parametric methods. A permutation test was performed to determine the LOD score threshold for the entire genome, with a significant level of 5% with 1,000 permutations. The peak marker position, LOD score, percentage of variation explained, and 95% confidence intervals using Bayesian methods were determined for each trait QTL.

### 2.6 Gene network analysis

A pathway enrichment analysis was performed for loci hotspots (> 3 QTL) where individual size-related traits were identified with overlapping confidence intervals and same peak marker position. Genes located within 700 Kb either side of the peak position were retrieved for enrichment analysis. This range was used to meet the criterion that candidate genes be within 3–4 cM of the peak position as determined by the size of the grape genome; therefore, a total of 1.4 Mb centered on the peak position was used (Cipriani et al., 2011; Hugalde et al., 2021). The VitisNet functional annotation of the genes within this 1.4 Mb region a Fisher’s test (p-value < 0.05 for gene network enrichment analysis was conducted (Grimplet et al., 2012; Alahakoon and Fennell, 2023; Osier, 2016).

## 3 Results

### 3.1 Temporal analysis of leaf traits in response to daylength

**Figure 2:**
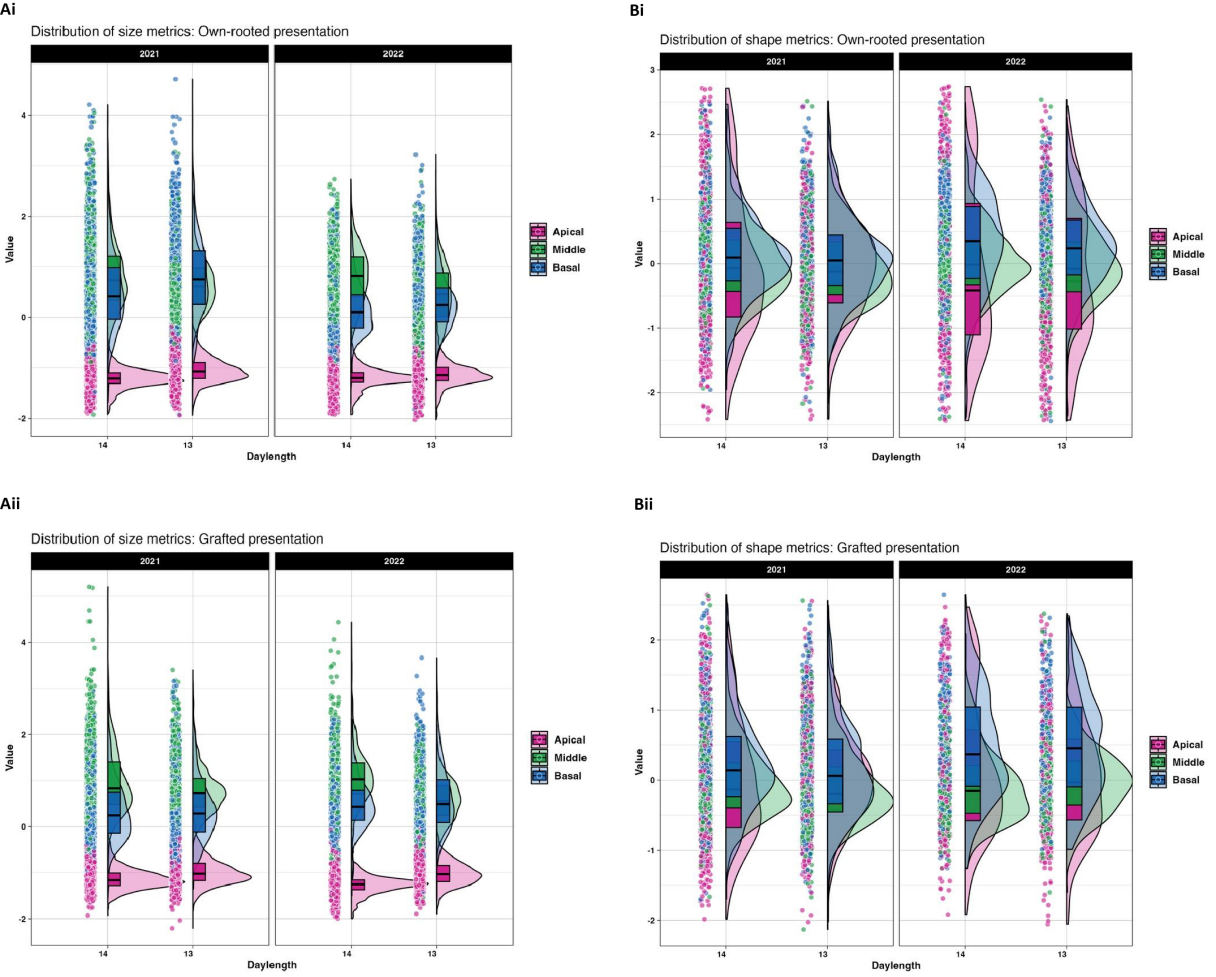
Leaf size and shape metrics for 14 h and 13 h daylengths for own-rooted (i) and grafted (ii) presentations of F1_Vruprip in 2021 and 2022. Size **(A)** and shape **(B)** metrics are illustrated by presenting Z-transformed data so results can be visualized on same scale across leaf positions for each vine presentation type (**i** own-rooted, **ii** grafted). Leaf position is noted as apical (pink), middle (green), and basal (blue). Each raincloud plot consists of a violin to illustrate the distribution density, a boxplot to show median and interquartile range, and individual data points (jittered) to highlight sample-level variation and outliers. The width of each violin (x axis) indicates the density of data points, with wider sections representing a higher frequency of data. Points beyond the violin plots represent individual outliers.

The sixteen different ampelographic phenotypes revealed different relationships according to leaf position and daylength as shown by Pearson correlation analysis (**Supplementary Figures 4**, **5**). Across all leaf positions, size related traits (area and length), had strong positive correlation with each other suggesting coordinated growth patterns in both presentation types. Veins traits correlated well with blade size, but their relationship with shape traits was low and highly variable across leaf positions. (**Figures 2A, B**) shows how leaf shape and size varied by position on the plant (apical, middle, and basal) under different daylengths in 2021 and 2022. In both own-rooted (**Figure 2Ai**) and grafted plants (**Figure 2Bi**), leaf position had a strong effect on leaf shape and size changes related to daylength. For both own-rooted F1_Vruprip and grafted vines, the apical leaf z transformed scores presented in **Figure 2** are consistently clustered in the lower range of values for 14 h and 13 h daylength. The 14 h was more similar for both presentations, while the 13 h violin plots showed a broader spread in the grafted presentation (**Figures 2Ai, Bi**). In contrast, the visualizations using shape z transformed scores showed a broad distribution and less influence of environmental factors (year, photoperiod) (**Figures 2Aii, Bii**).

**Figure 3:**
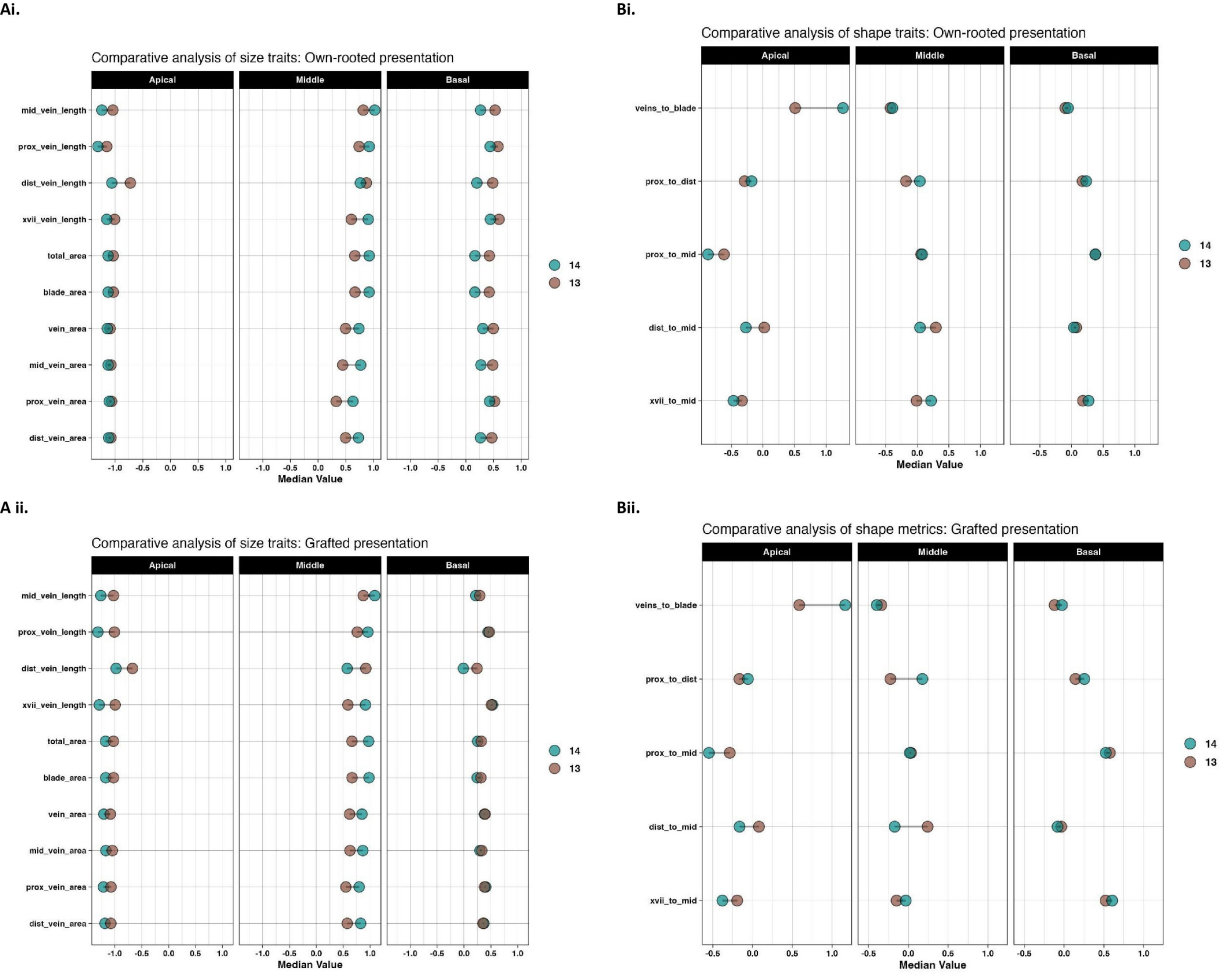
Median leaf size and shape across three leaf positions as influenced by daylength. Median values for size **(A)** and shape **(B)** traits for the own-rooted (i) and grafted (ii) presentations are shown for apical, middle, and basal leaf positions (left to right) at 14 (blue) and 13 h (brown).

The Kruskal-Wallis test indicated that daylength influenced the leaf size in own-rooted and grafted presentations (**Supplementary Table 2**). Shape related traits were not similarly affected in the own-rooted presentation but were noted in the middle and apical leaf positions in the grafted presentation. To further understand daylength influence on leaf morphology, individual traits were examined for each leaf position. (**Figures 3Ai, Aii**) showed that the size traits of own-rooted vines had similar patterns of change for the 13 h relative to the 14 h daylength. For the own-rooted presentation, the highest median leaf trait values were observed at 13 h for apical and basal leaves, in contrast to 14 h for middle leaf. In the grafted presentation, the scenario was different for the basal leaves, where 13 h and 14 h daylength measurements were similar for most traits, except for distal vein length. In the case of shape related traits, the shape changes in own-rooted and grafted presentation in 13 h relative to the 14 h were similar; however, the magnitude of change between the 14 h and 13 h daylength appeared to differ between the own-rooted and grafted presentations for some traits (**Figures 3Bi, Bii**). The median trait differences between daylengths were greater for the grafted presentation compared to the own-rooted for traits like proximal to distal vein length ratio and distal to mid vein length ratio in middle leaves.

### 3.2 Factors contributing to phenotypic variations in leaf morphology

**Figure 4:**
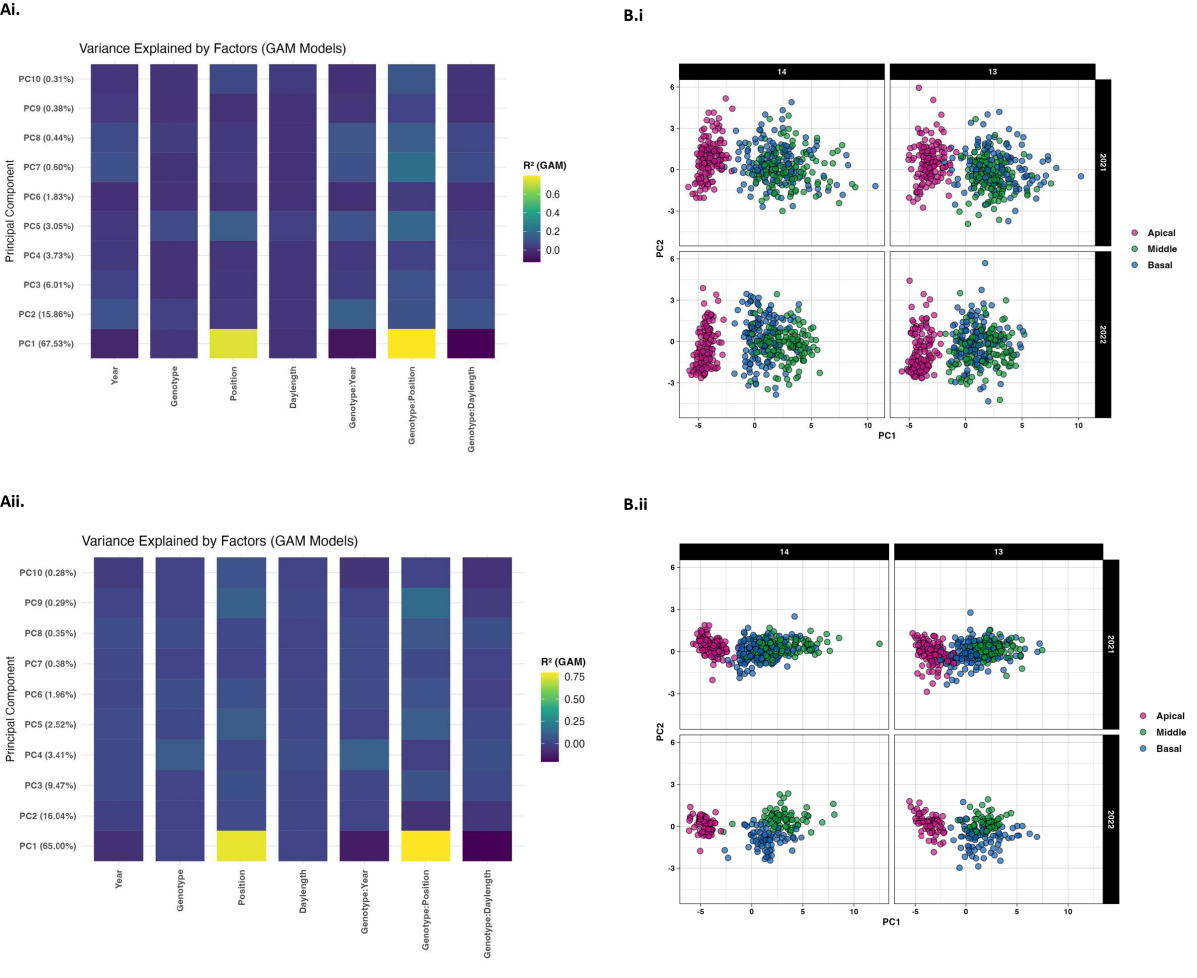
**(A)** Variance in principal components explained by main and interaction effects. The percentage of variability explained by the first 10 main components derived from leaf size and shape attributes are shown for own-rooted **(Ai)** and grafted presentation **(Aii)** for main factors (year, genotype, leaf position, daylength and interactions (genotype x year, genotype x leaf position, and genotype by position) y axis with portion of variance noted by color (0.2 (dark) to 0.8 (light). **(B)** PCA score scatterplots for leaf position relative to daylength. The distribution of the first two principal component scores for own-rooted **(Bi)** and grafted **(Bii)** at 14 and 13 h for apical (magenta), middle (green), and basal (blue) leaf position.

Principal component analysis performed on leaf morphological features, including size and shape attributes indicated that the first five PC components accounted for more than 95 percent of the overall variation observed in both own-rooted (**Figure 4Ai**) and grafted (**Figure 4Aii**) presentations. Year, genotype, position, daylength, and their interaction were determined to be the key factors in explaining the variance in leaf morphological phenotype (as represented by each PC score) using generalized additive models (GAM) for each PC score (**Figures 4Ai, Aii**). The findings showed that these factors had varying effects on distinct features of leaf morphology, with some aspects being more strongly influenced by one given factor than by another. According to the color-coded illustrations, the interaction effect between position and root genotype accounted for the highest percentage of variance contributing to PC1 for both own-rooted (4Ai) and grafted (4Aii) presentations.

The PCA biplot aids to further elucidate this relationship (**Figures 4Bi, Bii**) where the plots are categorized by daylength (14 h and 13 h), with distinct plots representing two distinct years (2021 in the top row and 2022 in the bottom row). Separation of the leaf positions across PC1 showed that each leaf position had its own unique morphological profile. There is a greater spread for the genotypes within each leaf position in both PC1 and PC2 for the own-rooted vines. Consistent patterns across the two years implied that the size and shape traits responded similarly to daylength differences. In both own-rooted (Bi) and grafted (Bii) presentations, the apical leaf group exhibited a distinct set of morphological characteristics that were detected by PC1 and PC2. The middle and basal leaf clusters overlapped in both 14 h and 13 h daylengths. When comparing presentation types, the points representing basal, middle, and apical leaf are more tightly clustered in grafted plants indicating lower genetic variability for all three leaf types. In contrast, the own-rooted plants show a broader range of variability representing the genetic differences within own-rooted leaves.

### 3.3 Differences in leaf morphology based on presentation type

**Figure 5:**
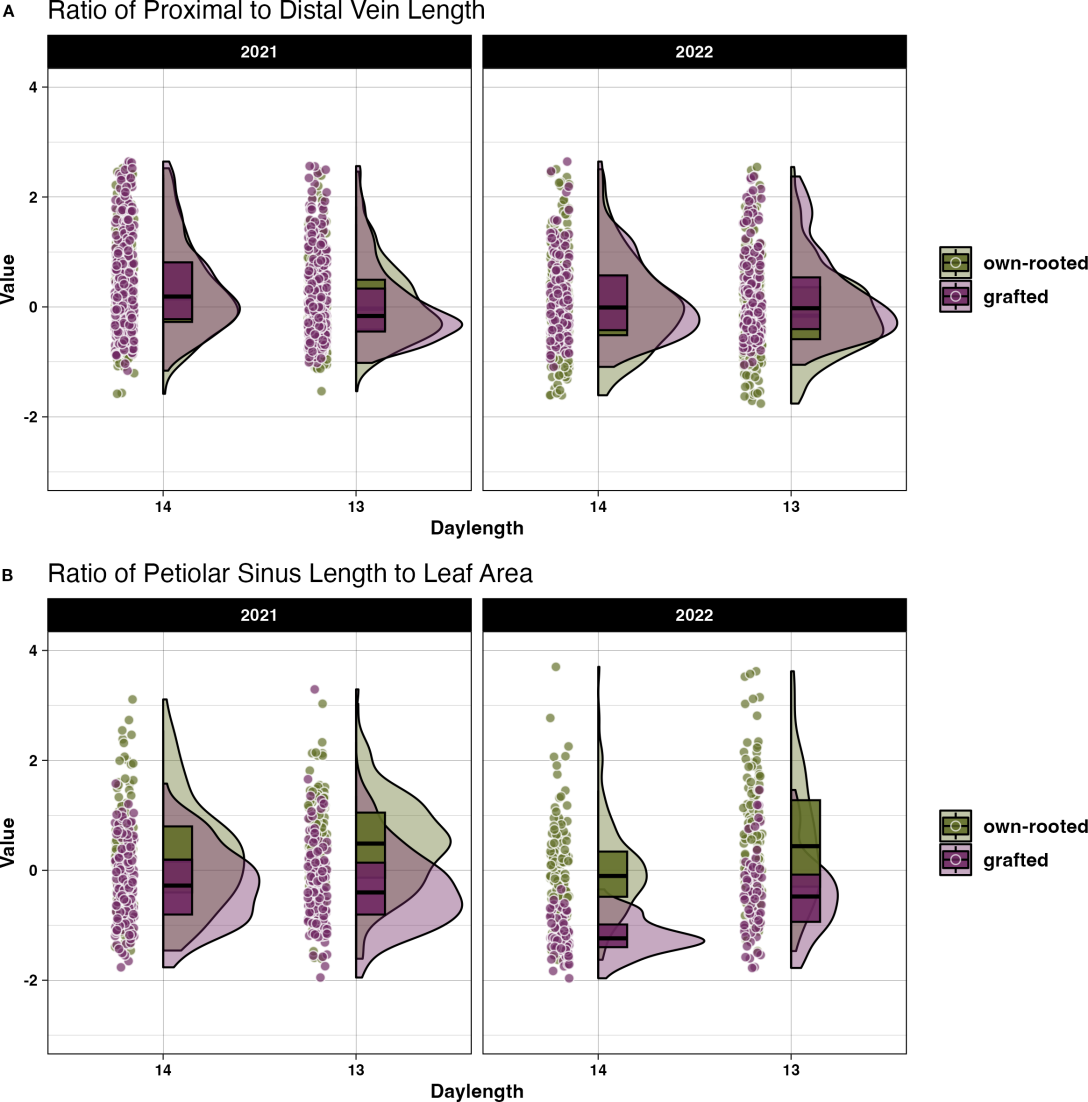
Differences in leaf shape morphology presented as z-transformed shape related measures for own-rooted and grafted vines. The distribution of the **(A)** proximal to mid vein length ratio and **(B)** ratio of petiolar sinus width to leaf area in own-rooted (green) and grafted vines (purple) for 14 and 13 h daylengths.

There was no significant difference in size related traits when compared across the own-rooted and grafted presentation types as shown by the Kruskal-Wallis test (**Supplementary Table 3**). However, for the shape related measures, two traits were significantly different between the presentations, the proximal to distal vein length ratio and petiolar sinus length to leaf area ratio. The difference is shown in the violin plots for 2021 and 2022 (**Figures 5A, B**). The median values for the proximal to distal vein length ratio the response to daylength was consistent between the own-rooted and grafted presentations in 2021 and 2022. The petiolar sinus length to leaf area ratio (**Figure 5B**) presented a notable difference in the pattern of variation, with the distribution of genotype samples being more spread out in the own-rooted presentation than in the grafted vines in both years.

### 3.4 Genetic linkage map

During map curation, 133 singleton and 901 monomorphic markers were found and no markers were identified as not grouping with a linkage group (LG). A total of 962 (48.1%) of the genotyped markers were anchored in 19 chromosomes spanning a total genetic distance of 1741 cM (**Supplementary Table 4**). The number of markers varied from 29 to 90 markers per LG, with an average of 50 markers anchored on each LG. The length of LG ranged from 71.1 cM (LG 15) to 154.4 cM (LG 14), with an average distance of 1.8 cM between markers (**Supplementary Table 3**). Genome-wide recombination rate of the integrated map was 0.24 cM/Mbp. The largest gap occurred on LG14 (33.3 cM) with the maximum gap on the other chromosomes between 6.7 to 17.5 cM. The genetic map covered 85% to 99% across the LG and there was strong collinearity between the genetic map when compared with the reference *V. vinifera* PN40024 12X.v2 genome providing a high genetic map quality (**Supplementary Figure 1**). No marker ordering errors were identified in this final map (**Supplementary Figure 2**) The F1_Vruprip rhAmpSeq was validated using sex type for a portion of the population grown in field using the binary QTL mapping method and subsequently the map was used for QTL analysis of leaf traits (Alahakoon et al., 2022).

### 3.5 QTL identified for size and shape attributes for vine presentation type

#### 3.5.1 Size attributes

In the own-rooted vines, 11 and 10 QTL were identified for 2021 and 2022, respectively (**Table 2**; **Supplementary Table 5**). In both years, the majority of QTL were found on chromosomes 14 and 19. Hotspots for size-related metrics were associated with marker rh19_472320 in basal leaves for 2021 and marker rh14_5687927 in apical leaves for 2022.

**Table 2:** Leaf size related trait quantitative trait loci (QTL) for own-rooted vines.

Year	Day	Trait	Ampelographic OIV designation	Leaf position	LOD	Peak position (cM)	Peak marker	Percent variance explained (%)
	Length							
2021	14	xvii_vein_length		middle	3.851	23.981	rh1_5782424	12.309
2021	14	prox_vein_length	OIV 603	middle	3.749	23.981	rh1_5782424	NA
2021	13	dist_vein_length	OIV 602	middle	4.597	45.882	rh7_18835505	14.613
2021	13	xvii_vein_length		basal	3.559	128.373	rh14_24366988	11.513
2021	13	mid_vein_length	OIV 601	basal	3.667	20.6	rh14_4941220	11.842
2021	14	dist_vein_length	OIV 602	middle	3.79	53.872	rh14_5687927	12.127
2021	13	dist_vein_area		basal	3.69	82.848	rh14_7777740	NA
2021	13	vein_area		basal	3.405	82.848	rh14_7777740	NA
2021	14	total_area		basal	4.021	4.249	rh19_472320	12.817
2021	14	prox_vein_area		basal	5.101	4.249	rh19_472320	15.972
2021	14	blade_area	OIV 065	basal	4.023	4.249	rh19_472320	12.824
2022	14	dist_vein_area		basal	3.82	32.968	rh7_10498469	12.302
2022	14	dist_vein_length	OIV 602	basal	5.283	83.397	rh8_19687962	16.603
2022	14	dist_vein_area		middle	3.592	4.478	rh8_301480	NA
2022	13	prox_vein_length	OIV 603	basal	3.468	80.48	rh10_16851267	NA
2022	13	total_area		apical	4.226	53.872	rh14_5687927	NA
2022	13	prox_vein_area		apical	4.828	53.872	rh14_5687927	NA
2022	13	vein_area		apical	4.261	53.872	rh14_5687927	NA
2022	13	blade_area	OIV 065	apical	4.122	53.872	rh14_5687927	NA
2022	13	prox_vein_area		basal	4.389	96.369	rh19_24344947	14.001
2022	13	dist_vein_length	OIV 602	basal	4.157	101.078	rh19_24632229	13.312

chr, chromosome; LOD, logarithm of odds, comparing the hypothesis of a QTL at a position versus that of no QTL % variation, the percent variation explained by each QTL; NA indicates non-parametric analysis. Confidence intervals and other details can be found in **Supplementary Table 4**.

A total of 27 QTL were detected in the grafted presentation with 23 of those QTL being identified for 2021 samples (**Table 3**; **Supplementary Table 6**). In 2021, QTL for area-related metrics were associated with rh2_17347640 and rh5_2193315 for middle and apical leaf positions. In addition, multiple QTL were found for rh16_5479328 locus and associated with total leaf area, blade area and proximal vein length. In 2022, only 4 QTL were found, with 2 each for the loci rh10_5150628 and rh12_5638 associated with the ratio traits proximal/distal vein area and length of proximal/xvii length, respectively.

**Table 3:** Leaf size related trait quantitative trait loci (QTL) for grafted vine presentation.

Year	Day length	Trait	Ampelographic OIV designation	Leaf position	LOD	Peak position (cM	Peak marker	Percent variance explained (%)
2021	14	total_area		middle	5.568	68.622	rh2_17347640	16.845
2021	14	prox_vein_area		middle	5.891	68.622	rh2_17347640	17.732
2021	14	vein_area		middle	4.523	68.622	rh2_17347640	13.916
2021	14	blade_area	OIV 065	middle	5.591	68.622	rh2_17347640	16.909
2021	14	prox_vein_length	OIV 603	middle	4.474	68.622	rh2_17347640	13.775
2021	13	mid_vein_length	OIV 601	apical	4.53	9.757	rh5_1508886	14.03
2021	13	total_area		apical	4.273	10.109	rh5_2193315	13.29
2021	13	prox_vein_area		apical	4.205	10.109	rh5_2193315	13.091
2021	13	mid_vein_area		apical	4.147	10.109	rh5_2193315	12.925
2021	13	vein_area		apical	4.42	10.109	rh5_2193315	13.714
2021	13	blade_area	OIV 065	apical	4.255	10.109	rh5_2193315	13.236
2021	13	prox_vein_length	OIV 603	apical	3.809	10.109	rh5_2193315	11.935
2021	13	dist_vein_area		apical	4.308	14.748	rh5_3398262	13.391
2021	13	mid_vein_area		basal	3.325	60.849	rh8_14638681	NA
2021	13	prox_vein_length	OIV 603	middle	3.962	2.668	rh11_1985173	12.386
2021	13	mid_vein_length	OIV 601	middle	3.551	2.668	rh11_1985173	NA
2021	13	vein_area		middle	3.947	9.027	rh11_3112858	NA
2021	13	prox_vein_length	OIV 603	middle	3.941	37.469	rh15_13822901	12.322
2021	13	xvii_vein_length		apical	3.757	16.218	rh16_1341063	11.783
2021	13	mid_vein_length	OIV 601	apical	4.322	18.561	rh16_2376878	13.431
2021	13	total_area		apical	4.272	23.97	rh16_5479328	13.288
2021	13	blade_area	OIV 065	apical	4.296	23.97	rh16_5479328	13.355
2021	13	prox_vein_length	OIV 603	apical	5.037	23.97	rh16_5479328	15.472
2022	14	prox_vein_area		apical	4.855	27.5	rh10_5150628	27.343
2022	14	dist_vein_area		apical	3.905	27.5	rh10_5150628	22.656
2022	13	prox_vein_length	OIV 603	basal	3.666	0	rh12_5638	21.163
2022	13	xvii_vein_length		basal	4.085	0	rh12_5638	23.274

chr, chromosome; LOD, logarithm of odds, comparing the hypothesis of a QTL at a position versus that of no QTL % variation, the percent variation explained by each QTL; NA indicates non-parametric analysis. Confidence intervals and other details can be found in **Supplementary Table 5**.

#### 3.5.2 Shape attributes

In contrast to the leaf size related traits, there were few QTLs identified for the leaf shape related traits. A total of 10 QTL were identified for own-rooted presentation with 5 QTL each for 2021 and 2022 (**Table 4**; **Supplementary Table 7**). The petiolar sinus to area trait was associated with QTL on different chromosomes in the own-rooted and grafted presentations with repeat hits on chromosome 1 for own-rooted and a QTL each on chromosome 2 and 4 for grafted presentation.

**Table 4:** Leaf shape related trait quantitative trait loci (QTL) for own-rooted and grafted presentations of F1 rootstock population.

Year	Day length	Trait	Ampelographic OIV designation	Leaf position	LOD	pos_peak(cM)	Peak marker	Percent variance explained (%)	
Own-rooted presentation									
2021	14	petiolar_sinus_to_area		middle	4.333	23.981	rh1_5782424	NA	
2021	13	petiolar_sinus_to_area		middle	5.325	45.139	rh1_9063579	16.724	
2021	14	veins_to_blade		middle	4.248	29.624	rh3_4315306	13.49	
2021	13	dist_to_mid	OIV 602/OIV 601	middle	4.412	45.882	rh7_18835505	NA	
2021	13	prox_to_mid	OIV 603/OIV 601	Basal	6.586	29.351	rh19_3058726	20.256	
2022	14	dist_to_mid	OIV 602/OIV 601	apical	3.555	34.682	rh7_11956846	NA	
2022	14	dist_to_mid	OIV 602/OIV 601	Basal	4.151	83.397	rh8_19687962	13.295	
2022	13	veins_to_blade		Basal	4.014	39.791	rh9_8899256	12.885	
2022	13	dist_to_mid	OIV 602/OIV 601	middle	3.769	73.723	rh10_15747189	NA	
2022	14	veins_to_blade		middle	3.806	153.923	rh14_28947831	12.26	
Grafted presentation									
2021	14	petiolar_sinus_to_area		middle	3.909	68.622	rh2_17347640	12.147	
2021	14	xvii_to_mid		apical	3.652	15.453	rh5_3885586	NA	
2021	13	veins_to_blade		apical	4.546	23.97	rh16_5479328	NA	
2021	13	xvii_to_mid		apical	19.632	95.304	rh19_24285394	48.063	
2022	13	petiolar_sinus_to_area		middle	3.636	16.432	rh4_3661349	21.007	

chr, chromosome; LOD, logarithm of odds, comparing the hypothesis of a QTL at a position versus that of no QTL % variation, the percent variation explained by each QTL; NA indicates non-parametric analysis. Confidence intervals and other details can be found in **Supplementary Table 6**.

### 3.6 Identification of pathways associated with QTL hotspots governing size-related traits

Five QTL hotspots, two in the own-rooted (rh14_5687927, rh19_472320) and three in grafted (rh2_17347640, rh5_2193315, and rh16_5479328) containing three or greater collocated QTL with identical peak markers were identified and pathway enrichment analyzed (**Table 5**). The QTL regions for the own-rooted QTL on chromosomes 14 and 19 identified 11 and 12 enriched pathways for DNA replication, amino acid metabolism, and transcription factors (**Supplementary Table 8**). In the grafted presentation there were eight, five, and 11 enriched pathways for hotspots on chromosomes eight, five, and 11 respectively (**Supplementary Table 8**). Transporters, transcription factors, and carbohydrate metabolism were found in these enriched pathways. The total lists of the genes analyzed for pathway enrichment can be found in **Supplementary Table 9**.

**Table 5:** Size-related morphological QTL hotspot pathway enrichment.

QTL peak marker	Presentation	Size-related trait	Significant pathways	Pathway summary
*rh14_5687927*	Own-rooted	four area and one length related traits in apical and middle leaf	11	Aminoacid metabolism, DNA replication, Cellular processes
*rh19_472320*	Own-rooted	three area related trait in basal leaf	11	DNA replication, Signal transduction, TF
*rh2_17347640*	Grafted	four area and one length related traits in middle leaf	8	Carbohydrate metabolism, Transporters, TF
*rh5_2193315*	Grafted	five area and one length related traits in apical leaf	5	Carbohydrate metabolism, Transporters, TF
*rh16_5479328*	Grafted	two area and one length related traits in apical leaf	4	Aminoacid metabolism, Cell growth, Cellular processes

Complete listing of significant pathways and contributing genes are identified in **Supplementary Tables 7**, **8**. Complete gene lists analyzed are identified in **Supplementary Table 10**.

Enriched pathways, including galactose metabolism, protein export, accessory factors involved in transport, BES1, and FHA, occurred repeatedly in multiple QTL regions. The cumulative enriched pathway results from all five QTL hotspot regions are presented in **Supplementary Table 8**. A total of five distinct enriched pathways associated with amino acid metabolism were identified. Various processes, including DNA replication, thylakoid targeting pathway, cell cycle, and RNA polymerase, were identified regarding protein modifications and cellular processes and growth. A collective of five distinct enriched pathways were identified regarding transport mechanisms. In addition, twelve distinct transcription factors were identified, including BES1, GNAT, FHA, and CAMTA (**Supplementary Table 8**). Signal transduction-related enriched pathways for brassinosteriod, auxin, and ABA signaling were found. Likewise, a total of six distinct pathways for carbohydrate and lipid metabolism were identified.

## 4 Discussion

Previous studies of the genetic determination of leaf morphology are single year studies of F1 populations that center on differences from a single shoot position relative to the leaf shape traits of leaf vein angles and lobing in the progeny (Welter et al., 2007; Demmings et al., 2019). This study was conducted in a non-lobed population in 2 years with three leaf positions to monitor position and time/daylength potential impacts.

### 4.1 Leaf size and shape traits change significantly with decrease in daylength

Daylength varies by latitude and season and is a well-known regulator of plant development and physiology (Jackson, 2009). In *Arabidopsis thaliana*, daylength reductions have been reported to reduce the absolute rate of leaf expansion, suggesting that photoperiod has a direct influence on the dynamics of leaf development (Cookson et al., 2007). The progression or temporal shift related to leaf size and shape in response to a decrease in photoperiod in this study varied with the i) type of leaf trait (size or shape) and ii) leaf position. Visual examination of the leaves suggested that the shape related traits changes were less variable than size-related traits across daylength. While leaf shape was more conserved, it still exhibited variability within certain bounds to changing daylength conditions as evidenced by the results of Kruskal-Wallis test. Specifically, the middle leaf differed significantly when compared across daylengths for both years in the grafted presentation and for 2022 in the own-rooted presentation. Similarly, in *Vitis* hybrid ‘Marquette’ grafted to five commercial rootstocks: 1103 Paulsen (1103P), 3309 Couderc (3309C), Teleki 5C (5C), Freedom (FREE), Selection Oppenheim 4 (SO4), as well as homografted controls, physiological assessments using a portable photosynthesis system (LI-6800; LI-COR Biosciences, Lincoln, NE, USA) showed a decrease in the net assimilation rate (A), the maximum rate of carboxylation (Vcmax), and the maximum electron transport rate (Jmax) in middle leaf, when daylength decreased from 15 to 14 hours (Sharma et al., 2024a, Sharma et al., 2024b). These three studies suggest that the middle leaf is more developmentally plastic in terms of structural features and responds to environmental influences to a greater extent. In contrast, the basal leaf remained consistently non-significant for both own-rooted and grafted presentations, these leaves were formed before the leaf measurement began; therefore, would not be expected to show morphological shifts during the study, although slight variations could occur as basal leaves can be shed as they age. This finding aligns with a previous *V. riparia* study where oldest leaf was found to be invariant likely because they focused on maximizing early season growth or are controlled by genetics that limit changes in their shape and size (Chitwood et al., 2016b; Baumgartner et al., 2020). In contrast, intermediate leaves showed greater phenotypic plasticity and were interpreted to have strongest climate signals (Chitwood et al., 2016b). The major takeaway is that there was significant difference in leaf size and shape traits across different daylengths. However, the underlying drivers of this phenotypic plasticity are complex and could be related to combination of environmental cue like daylength, allometry (varying growth rates across an organ) or heteroblasty (trait differences based on successive nodes) or combination of these (Baumgartner et al., 2020; Evans, 1972; Spriggs et al., 2018; Chitwood et al., 2014a; Coleman et al., 1994).

### 4.2 Leaf morphological differences are contributed by genetic and developmental effects

To develop a greater understanding of the major factors contributing to variation in leaf morphology (shape and size) several factors were considered i.e., i) genotype effect ii) environmental effect (year, daylength) and iii) developmental effect (leaf position). These studies suggested that interaction between genotype and position were major contributors to the variation in leaf morphological traits. PC1 separated the root genotypes based on leaf positions, where apical leaf was separated from middle and basal leaf in all cases (multiple daylength and year) (**Figures 4Aii, Bii**). Moreover, the variation between the data points along y axis showed the variation within specific leaf positions for genotypes. This y axis or PC2 dimension primarily showed how genotypes varied at particular position for given presentation type. Klein et al. (2017), leaf morphometrics study illustrates differences between *V. rupestris* Sheele and *V. riparia* Michx. with linear discriminant analysis and was able to predict their leaf shape with accuracy of >98%. Our study utilized two F1 presentations derived from these two species, and the observed differences observed in morphology could be attributed to the genetic contribution of either parent. It is noted in the PC biplot that own-rooted had a greater distribution than that conferred to the common scion in the grafted presentation. This implied that the common scion ‘Marquette’ may have minimized variations within the grafted presentations. When comparing presentation types, only two traits i.e., proximal to distal vein length ratios and petiolar sinus to leaf area ratios were significantly different between own-rooted and grafted types (**Supplementary Table 3**). This indicated that the genetically different root systems conferred an influence on the common scion and affected the narrowness/width of leaves when measured perpendicular to midrib as well as the length of petiolar sinus. Pearson correlation of the 2023 pruning weights (year following leaf study) with the leaf total area and blade area (2022) of this study showed significant correlation and provide further support for the rootstock influence on leaf morphology subsequently impacting the scion shoots (**Supplementary Table 10**). Rootstock-scion interaction is a complex and dynamic phenomenon in grapevines, and the extent to which rootstock influences scion remains an area of active research. Previous studies in grapevines have demonstrated that rootstock has a direct influence on elemental compositions of leaf, its shape, as well as physiological responses (Migicovsky et al., 2019; Harris et al., 2021; Sharma et al., 2024a, Sharma et al., 2024b). For example, research on *V. vinifera* 'Italia' grapes comparing ungrafted vines and grafted vines (two distinct rootstocks) under two irrigation settings revealed that the leaf area was significantly influenced by rootstock-by-irrigation interaction (Sabir, 2016). Like these findings, our study identified certain morphological features associated with rootstock effect. This suggests broader implications of rootstock selection in viticulture aiming for vine performance improvement, optimizing canopy structure and tailoring growth responses based on production goals.

### 4.3 QTL hotspots highlight leaf-growth related pathways

The QTL for the own-rooted and grafted presentations provided different genetic loci associations. For the own-rooted presentation, QTL were frequently identified on chromosomes 8,14, and 19 (**Table 2**). These QTL explained from 12-17% variation for a given phenotype. In contrast, the QTL for the grafted presentation of the F1 population were mostly identified in chromosome 2, 5 and 16 and explained 11- 27% total phenotypic variation (**Table 3**). For shape related trait measures, there were no hotspots from co-localized traits, but there was a QTL with exceptionally higher LOD score 19.63 (**Table 4**). This QTL was responsible for around 48% of phenotypic variation in the apical leaf of grafted presentation and was associated with chromosome 19. This is a significant foundation for further investigation as this suggests a strong potential leaf morphometric phenotype.

The QTL hotspots regions selected from size traits identified pathways known to be associated with leaf growth and development (**Table 5**). The enriched pathways identified under QTL hotpot regions were associated with development related processes like cell cycle, thylakoid targeting pathway, DNA replication as well as protein export. Similarly, enriched carbohydrate-related pathways (starch, sucrose, galactose) and amino acid (glutamate, cysteine, lysine, tyrosine) related metabolic pathways were identified. Leaf size increase or other leaf growth characters requires substantial demand of carbon skeleton molecules to build new structures like cellulose and hemicellulose in cell walls. It also involves the use of photosynthetic carbon to initiate various energy consuming mechanisms like cell cycle and protein synthesis. The leaf growth rate is correlated with activity of carbon metabolism enzymes or level of carbon metabolites (Sulpice et al., 2009; Fichtner et al., 1993; Cross et al., 2006). Similarly, amino acids serve as precursor for the synthesis of variety of compounds necessary for plant developments like chlorophyll, nucleotides, and secondary metabolites (Tegeder, 2012). The rh19_472320 QTL hotspot had one of the most interesting characteristics with three significant interrelated pathways Brassinosteroid_biosynthesis vv10905, Brassinosteroids_signaling vv30005, and Brassinosteroid-activated BRI1-EMS-Supressor transcription factors vv60010BES1. Brassinosteroids (BR) are growth-promoting hormones known to have role in plant cell expansion, elongation, and proliferation impacting overall leaf size (Zhang et al., 2021; Clément et al., 2018; Zhiponova et al., 2013; Gudesblat and Russinova 2011). One other hormone signaling process identified was ABA signaling. ABA is found to impact both expansive and structural growth inhibiting cell and plastid division (Pantin et al., 2012; Wang et al., 1998; Galpaz et al., 2008). ABA not only regulates stomata and aquaporins controlling incoming carbon dioxide, but it is also found to regulate carbon metabolism at enzymatic level at both transcriptional and post transcriptional scenarios (Zhu et al., 2011). BES1 transcription factor, which was identified in two hotspot peak regions, is known to activate BRinduced gene expression in plants (Yin et al., 2005). The other repeatedly occurring transcription factor was fork-head transcription factor (FH). FHA or fork-head associated proteins are known to be associated with multiple functions in regulating plant organ development, signal transduction, hormone response, and DNA damage repair in Arabidopsis (Wang, 2023). In summary, findings of leaf growth associated pathways reveal that the identified QTL are important and need to be further validated in natural environment settings.

## 5 Conclusion

This research examined phenotypic and genetic basis of leaf size and shape differentiation within two presentations of an F1 rootstock population (*V. rupestris* Scheele ‘B38’ X *V. riparia* Michx ‘HP1’). Previous genetic studies have focused on shape related traits such as leaf lobing in similar aged leaves from own rooted grapevine populations. In contrast this study explored the developmental factor of leaf position and environmental factors (year, daylength) in an own rooted and grafted rootstock population to map the genetic basis of leaf size and shape. Genetic analysis identified five hotspots associated with size-related attributes. The identification of multiple leaf-growth-associated pathways in these hotspot regions defined the connection between genetics and phenotypic traits. Although there were no QTL hotspots for shape related traits, several explained a larger amount of the phenotypic variation in leaf morphology. It is notable that rootstock effects were conferred to the scion as determined by three distinct size related traits that influenced vein and blade area of the leaves. Rootstock-scion interaction is a dynamic phenomenon and identifications of morphological features impacted by rootstock provide opportunity for new vine combinations. Specifically, these QTL regions can be used to identify molecular networks associated with leaf growth regulation to design grapevine combinations with biomass efficiency and adaptability to withstand rapid changing climate.

## Data Availability

The datasets presented in this study can be found in online repositories. The names of the repository/repositories and accession number(s) can be found below: https://doi.org.10.62812/TWTH2085, https://doi.org.10.62812/EBZX2792.
