# Supplementary material for: Genetic regulation of leaf morphology in own-rooted and grafted vines of an F1 rootstock population
Source: Front Plant Sci. 2025 Oct 23;16:1625453. doi: 10.3389/fpls.2025.1625453 (PMC12588919; doi:10.3389/fpls.2025.1625453)
Supplement: Supplementary file 1 [file Supplementaryfile1.docx]

Supplementary Material

## Supplementary Figures

##
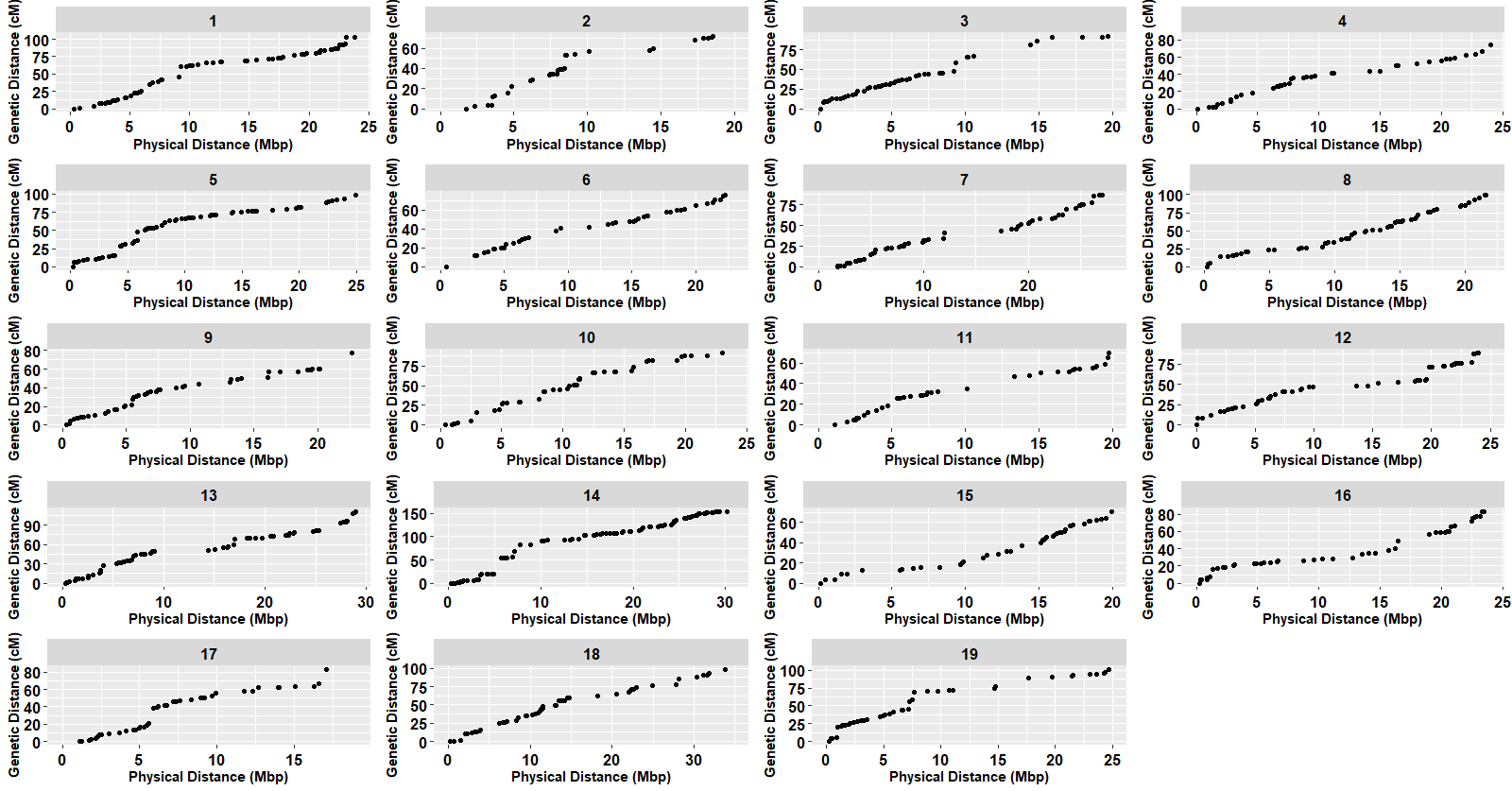


## Supplementary Figure 1. Collinearity between *V. vinifera* PN40024 V2 reference genome physical positions and genetic positions of the F1_Vruprip_rhAmpSeq map.for all 19 chromosomes.

**
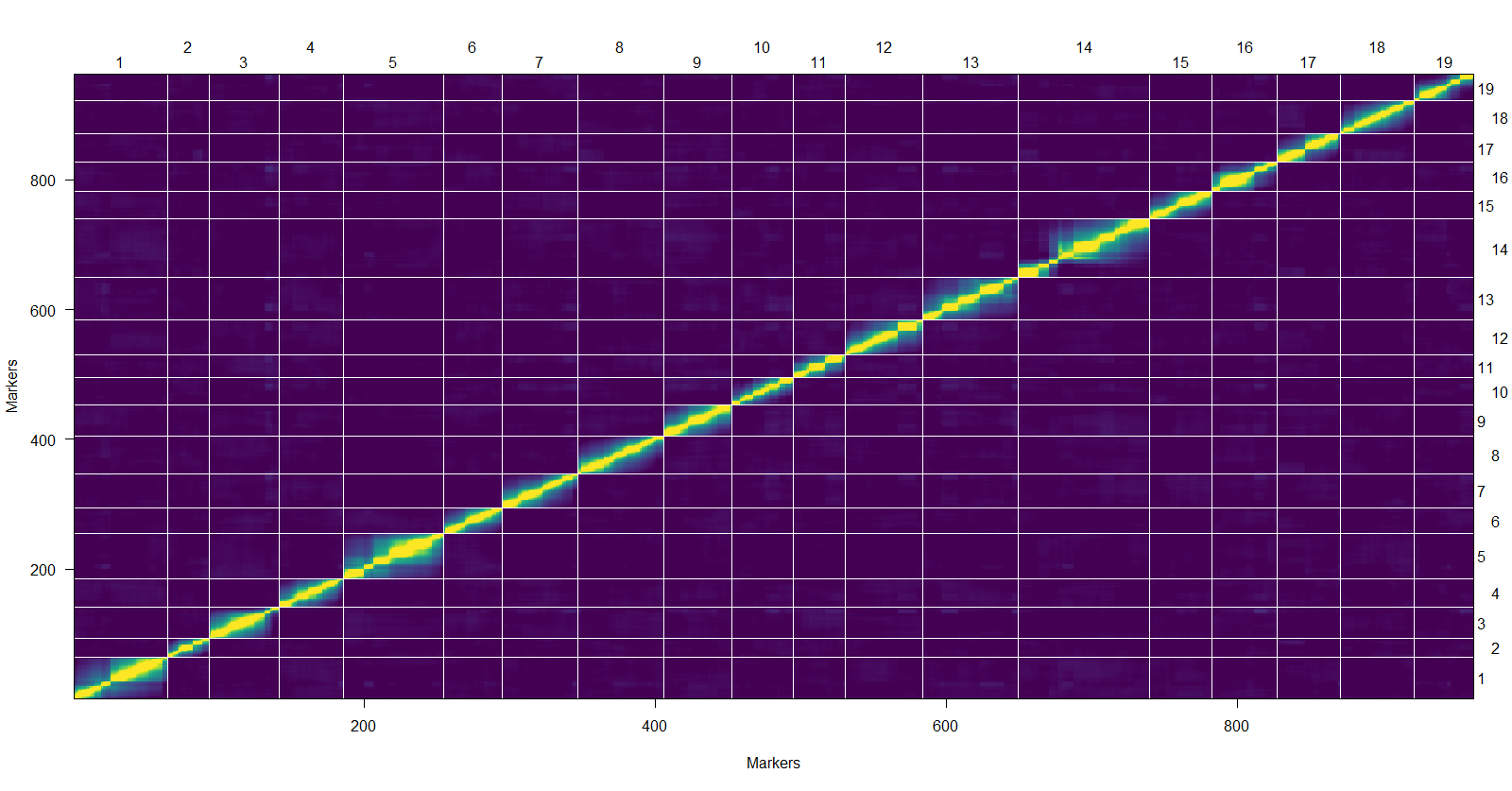
**

**Supplementary Figure 2.** Pair-wise recombination fractions and LOD of the VRS-F2 genetic map. Vertical and horizontal lines indicate the borders of the linkage groups. The estimated recombination fractions (r) between markers are in the upper left and the LOD are in the lower right of each linkage group rectangle. High correlation between markers indicates marker linkage (yellow, low ˆr or high LOD) and blue (high ˆr or low LOD) represents low correlation values indicating unlinked markers.


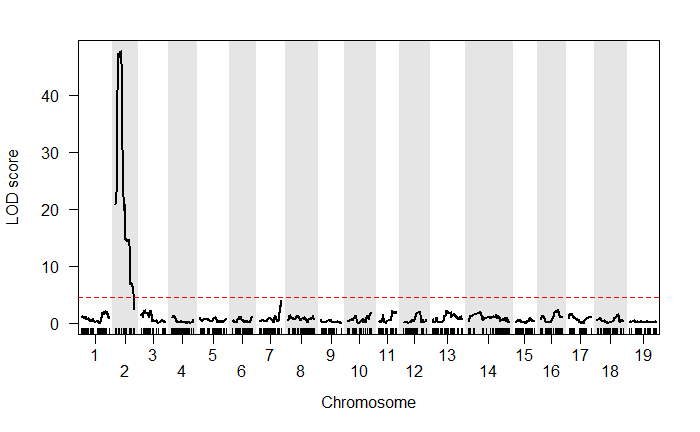


**Supplementary Figure 3.** Map validation with flower type (pistillate, staminate).


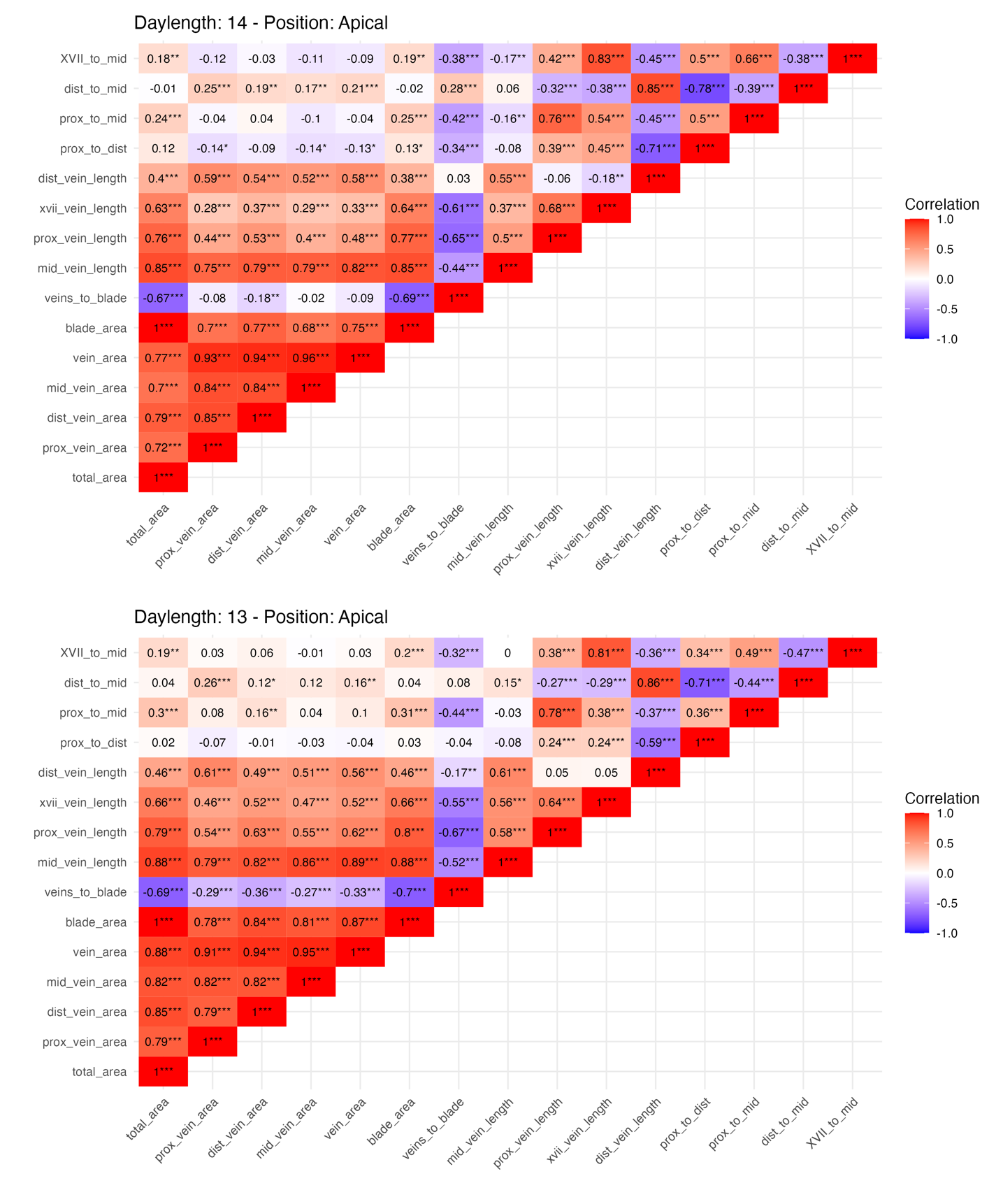


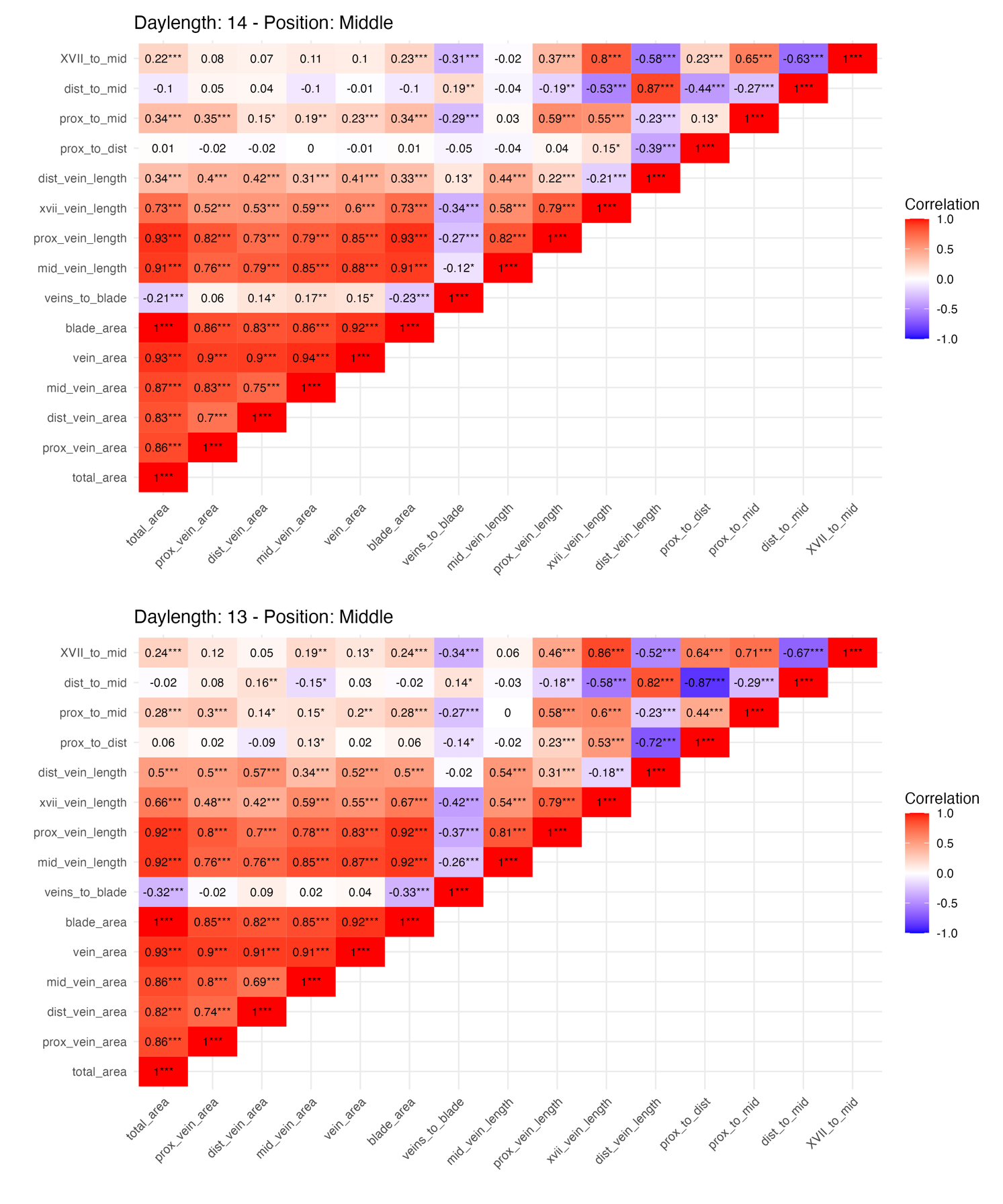


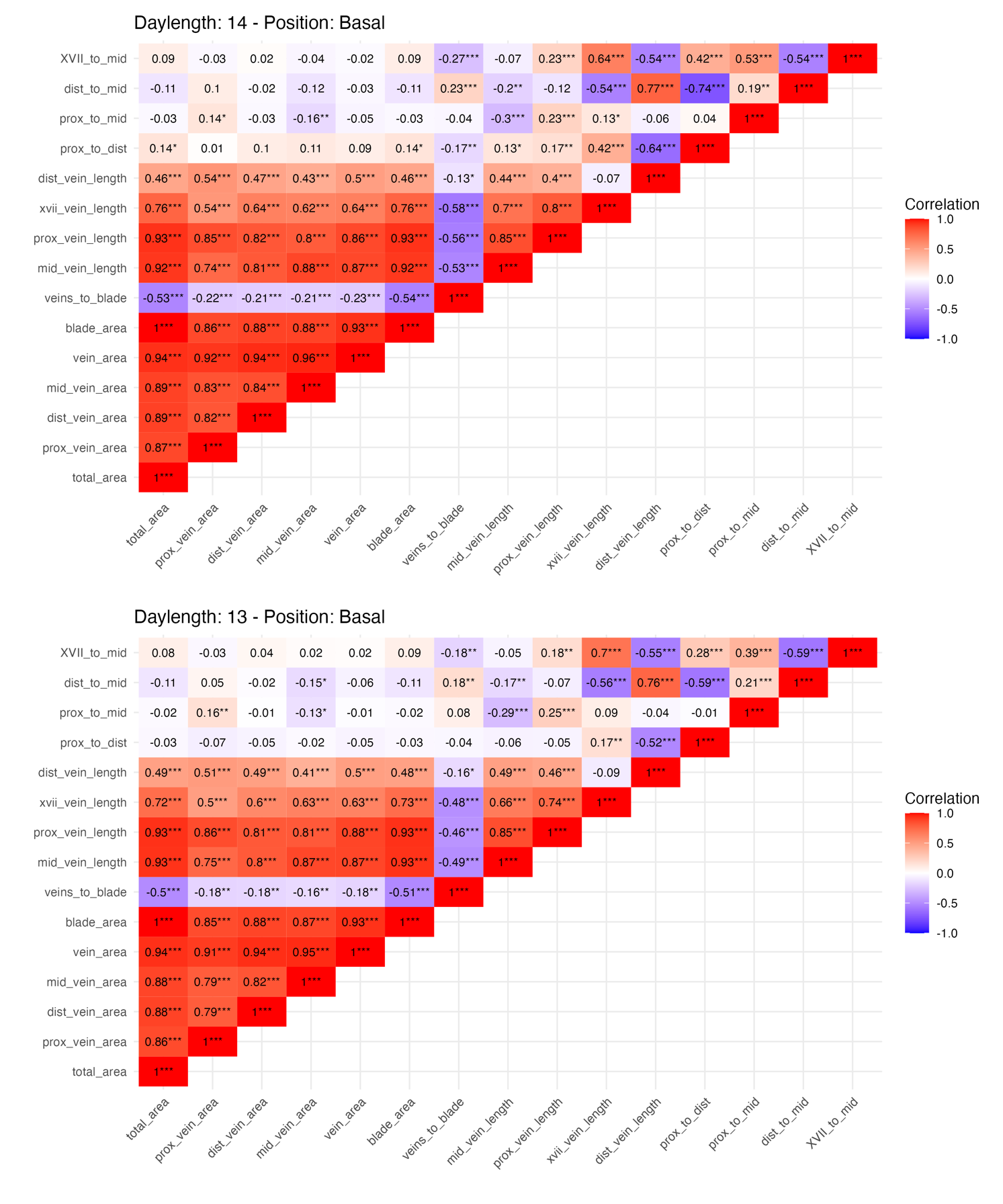


**Supplementary Figure 4.** Pearson correlation matrix of leaf size- and shape-related traits in the own-rooted grapevine population across three leaf positions: (i) apical, (ii) middle, and (iii) basal leaves. Each cell displays the Pearson correlation coefficient (r) between trait pairs. Statistically significant correlations (p ≤ 0.05) are denoted by asterisks (*), with increasing significance indicated as follows: *p ≤ 0.05, **p ≤ 0.01, ***p ≤ 0.001. Positive correlations are shown in red, negative correlations in blue, with intensity corresponding to the strength of the association.


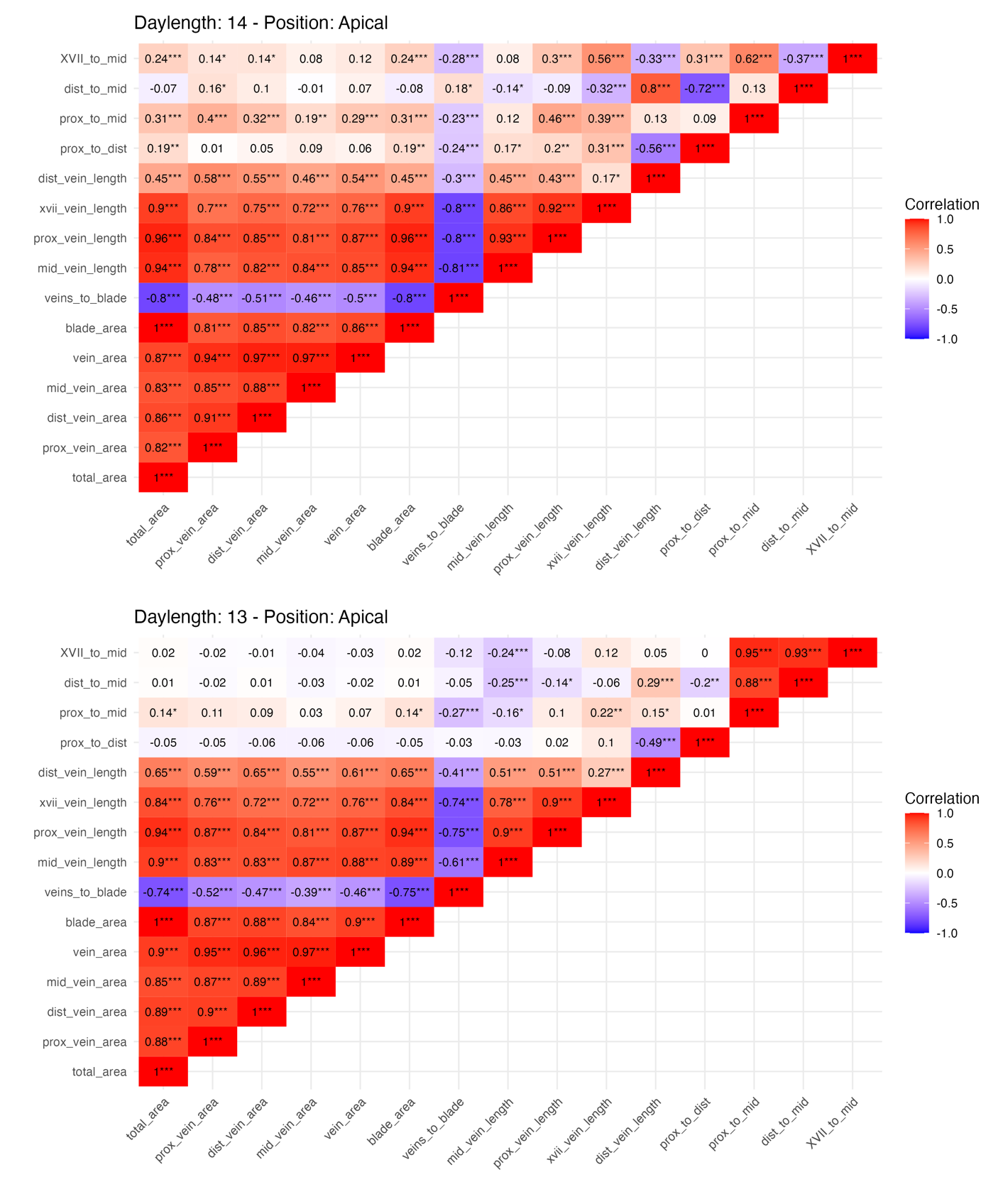


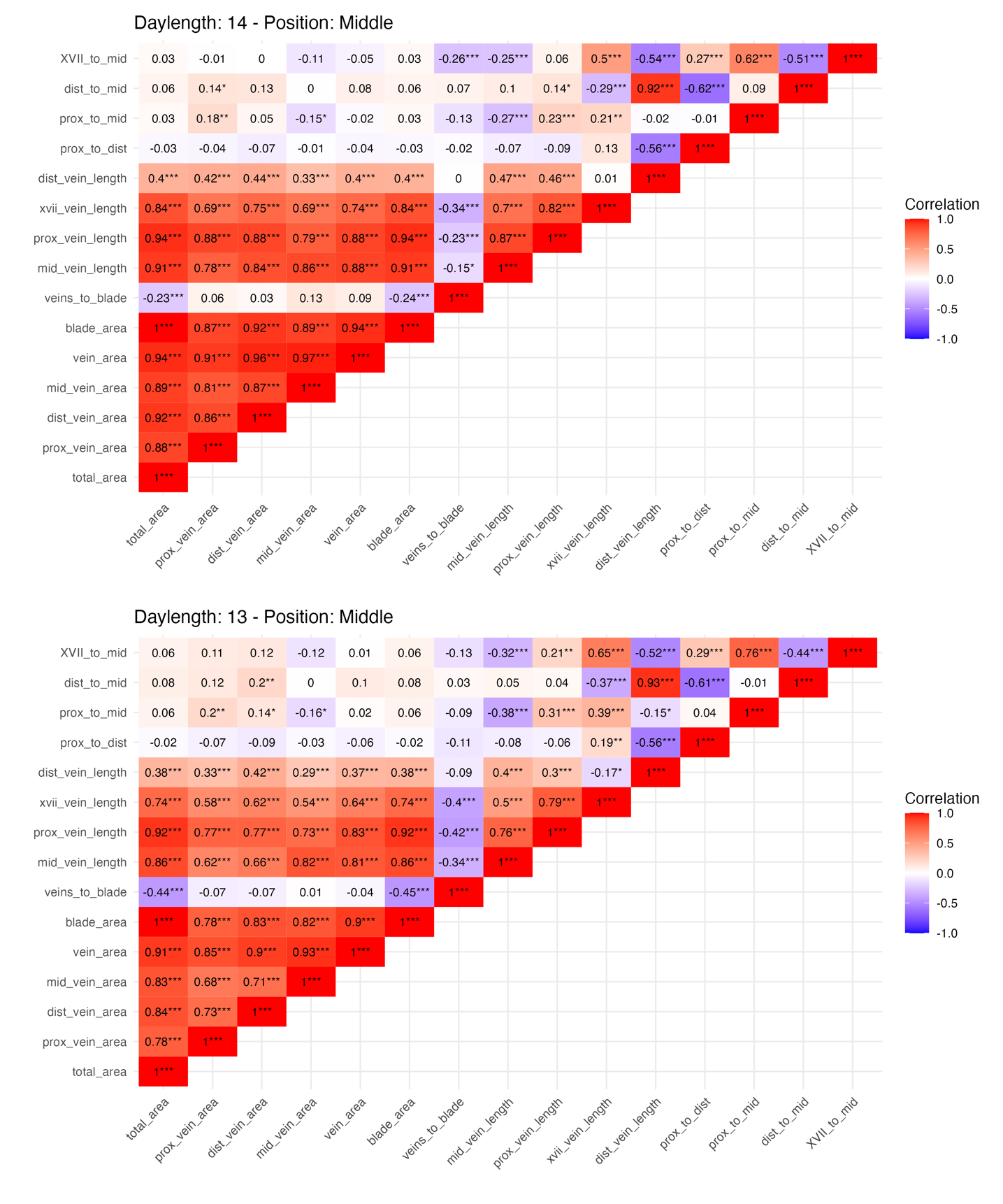


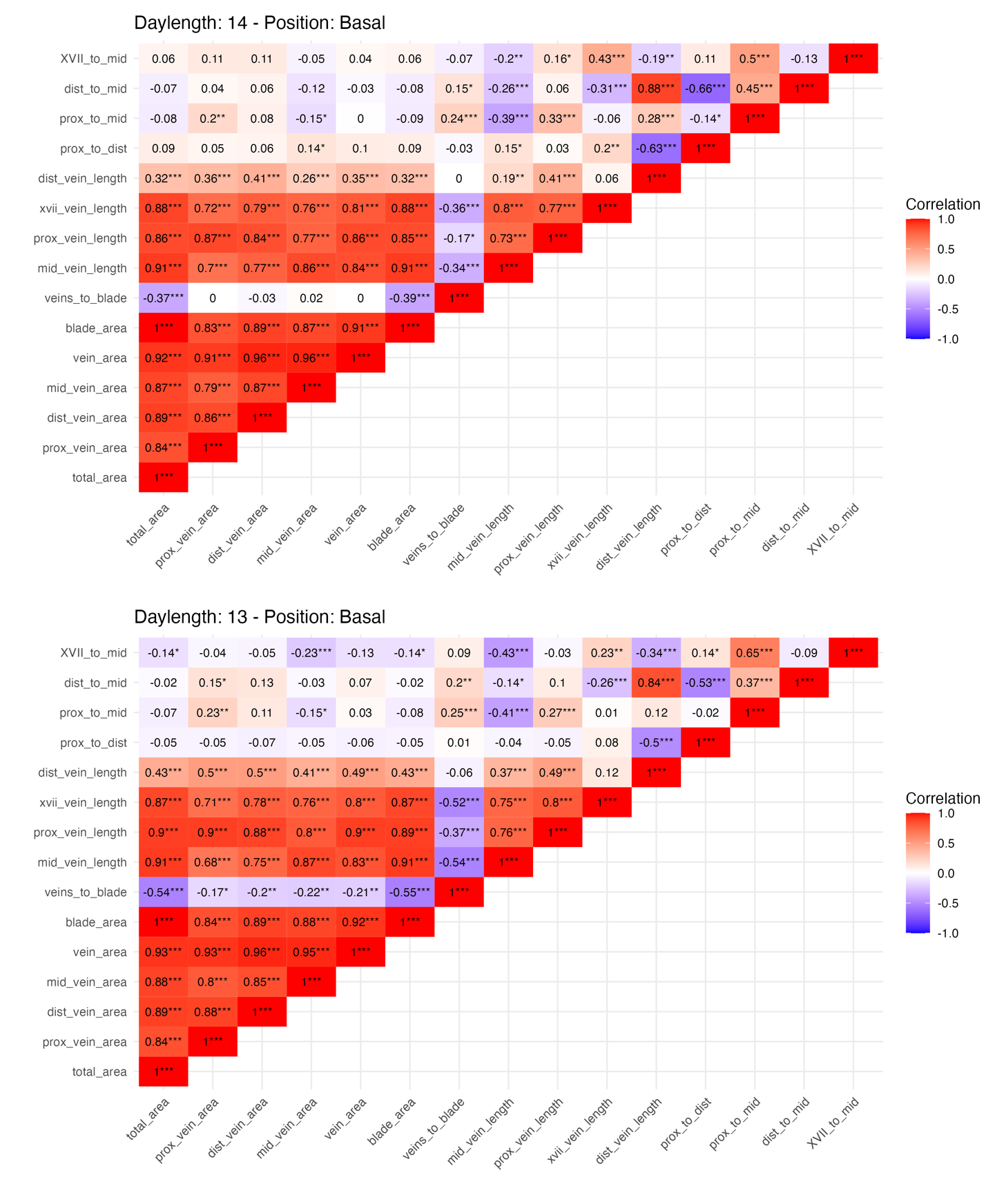


**Supplementary Figure 5.** Pearson correlation matrix of leaf size- and shape-related traits in the grafted grapevine population across three leaf positions: (i) apical, (ii) middle, and (iii) basal leaves. Each cell displays the Pearson correlation coefficient (r) between trait pairs. Statistically significant correlations (p ≤ 0.05) are denoted by asterisks (*), with increasing significance indicated as follows: *p ≤ 0.05, **p ≤ 0.01, ***p ≤ 0.001. Positive correlations are shown in red, negative correlations in blue, with intensity corresponding to the strength of the association.
